# The Dilemma of Derelict Gear

**DOI:** 10.1038/srep19671

**Published:** 2016-01-21

**Authors:** A. M. Scheld, D. M. Bilkovic, K. J. Havens

**Affiliations:** 1 Virginia Institute of Marine Science, College of William & Mary, 1375 Greate Rd., Gloucester Point, Virginia, 23062 U.S.A..

## Abstract

Every year, millions of pots and traps are lost in crustacean fisheries around the world. Derelict fishing gear has been found to produce several harmful environmental and ecological effects, however socioeconomic consequences have been investigated less frequently. We analyze the economic effects of a substantial derelict pot removal program in the largest estuary of the United States, the Chesapeake Bay. By combining spatially resolved data on derelict pot removals with commercial blue crab (*Callinectes sapidus*) harvests and effort, we show that removing 34,408 derelict pots led to significant gains in gear efficiency and an additional 13,504 MT in harvest valued at US $21.3 million—a 27% increase above that which would have occurred without removals. Model results are extended to a global analysis where it is seen that US $831 million in landings could be recovered annually by removing less than 10% of the derelict pots and traps from major crustacean fisheries. An unfortunate common pool externality, the degradation of marine environments is detrimental not only to marine organisms and biota, but also to those individuals and communities whose livelihoods and culture depend on profitable and sustainable marine resource use.

The financial ruin of commercial fisheries, thought to squander US $50 billion in economic benefits annually^1^, has long been attributed to the common-pool nature of the resource^2^. Much like the 19^th^ century dilemma of overgrazing common pasture, economically rational, self-interested fishers reap the benefit of their labors individually while sharing in the cost of a depleted stock. Unfortunately for the fisher, a common fish stock is not all that is shared. The environment in which harvest occurs is also a common resource, whose collective maintenance or degradation affects individual efficiencies and economic returns. Across many of the world’s oceans and waterways, Hardin’s tragedy^3^ is multifaceted and complex.

Growth in global economies, together with the increasing use of long-lasting synthetic materials, has led to significant concerns surrounding marine debris^4^,^5^. Derelict fishing gear—the nets, lines, traps, and other recreational or commercial fishing equipment that has been lost, abandoned, or otherwise discarded^6^,^7^—is a major source of marine debris which has been charged with damaging sensitive habitats^8^, creating navigational hazards^9^, as well as reducing populations of target and non-target species^10^,^11^,^12^,^13^,^14^,^15^,^16^. Derelict gear may also compromise the economic vitality of fishery dependent businesses and communities as it competes with active gear and acts as a deterrent or distraction to target stocks, generating production inefficiencies which erode industry profits and inhibit commercial fishery success. These purely economic costs can be considered independent of the negative biological effects which might result from the continual capture of animals by derelict gear, termed ‘ghost fishing’. That is, derelict gear may impose an economic cost, in terms of reduced gear efficiency, even in cases of little to no ghost fishing mortality.

The United States Atlantic blue crab commercial fishery lands over 77,000 metric tons (MT) worth US $150–200 million annually^17^. In the Chesapeake Bay, which accounts for nearly half of all US blue crab landings, it is thought that 20% of the approximately 800,000 fished hard crab pots become derelict each year^15^. Derelict pots may self-bait and ghost fish for several years^8^ and experiments in the Chesapeake indicate structural integrity is generally maintained for two years or more^18^. Blue crabs are known to be attracted to pots as bottom structure whether or not any bait is present^18^,^19^,^20^, and it has also commonly been observed that crustaceans enter and leave pots frequently, with retention rates varying according to pot design and intra and inter-species interactions^20^,^21^,^22^,^23^,^24^. In the United States’ largest estuary, conservative estimates would suggest over 300,000 derelict pots are continually attracting, capturing, and possibly even killing, blue crab and other species (Fig. 1). As a result, active gear efficiency, harvests, and resource rents may be reduced considerably.

In 2008, following many years of declining harvests, the Chesapeake Bay blue crab industry was declared a commercial fishery failure by the US Department of Commerce, unleashing $30 million in disaster relief. A small portion of these funds was used to support the Virginia Marine Debris Location and Removal Program, a novel initiative in which commercial crabbers were hired during the winter closed fishing seasons to find, document, and remove derelict gear. The program proved to be a success, offering fishers an opportunity to earn supplemental income while also removing considerable amounts of marine debris and generating useful scientific data^25^. From 2008 to 2014, 34,408 derelict pots were removed (Supplementary Fig. S1). Throughout the removal program, harvests and gear efficiency were observed to increase dramatically (Supplementary Fig. S2).

## Results

### Chesapeake Bay

**Figure 2:**
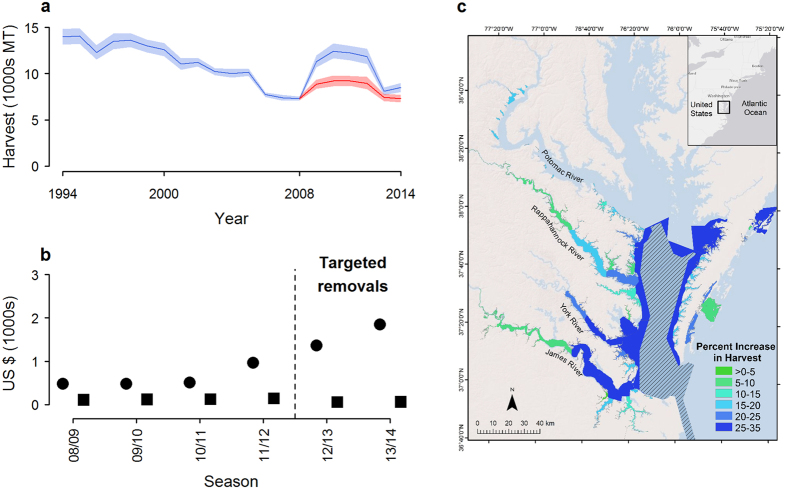
Economic effects of derelict pot removals. (**a**) 95% confidence region of Virginia blue crab harvest with (blue) and without (red) the Virginia Marine Debris Location and Removal Program. (**b**) Average benefits (circles) and costs (squares) per pot removed. Average benefits equal estimated total revenue increase divided by derelict pots removed. Average costs equal total compensation paid for removals divided by derelict pots removed. Vertical dashed line denotes start of removals from targeted hotspot areas. (**c**) Map of predicted harvest increases. Hatched area is a no-take crab sanctuary. Map created using Esri ArcGIS 10.0 ( http://www.esri.com/software/arcgis).

A spatially explicit harvest model was used to predict harvests under two scenarios: actual removals and a counterfactual of zero removals (i.e., what *would* have been harvested had no derelict pots been removed). In the counterfactual it was assumed that the observed increases in blue crab abundance were the result of contemporaneous conservation measures or advantageous environmental conditions, allowing identification of harvest increases arising solely from reduced gear competition. Model results indicate that removing only 9% of the derelict gear in Virginia waters increased harvests by 13,504 MT (*SE* = 1,660), or 27% (Fig. 2a). Harvest increases resulting from gear efficiency improvements averaged 0.22 kg/pot (*SE* = 0.03). During the removal effort, each actively fished pot was harvesting an additional blue crab on every pull—crab which would have been captured or attracted to the now absent derelict gear.

Without the removal program, US $21.3 million in blue crab revenues would have been lost. These benefits far outweighed the program’s total cost of US $4.2 million. Derelict pot removals were found to be net beneficial in every year of the program, though the difference between average benefits and costs per pot removed was greatest during the last two seasons, when limited program funds were used to target derelict gear hotspots (Fig. 2b). During targeted removals, a small group of commercial crab fishers focused removal efforts in areas which regularly experience high rates of potting activity and gear loss. Removals from these areas were more effective, and in general, areas which regularly experience high levels of effort and harvest, such as the mouths of major tributaries, saw greater program benefits (Fig. 2c). Considerable spatial and temporal heterogeneity in program effects suggests area and time prioritization of removals can be successful in producing significant economic benefit. For example, a removal effort at 10% the scale of the actual program (i.e., 3,441 removals), but focused on only the ten most heavily fished sites, would have increased harvests by 8,144 MT (*SE* = 1,328), or about 60% the improvement seen following the full removal program, *ceteris paribus*.

### Global Analysis

Derelict fishing gear is a global problem^16^. High rates of gear loss plague many of the world’s crustacean fisheries (Table 1) and, as a result, fishing traps and pots are thought to be one of the most common types of derelict gear worldwide^26^. Modern pots and traps are often constructed from rigid and durable materials^16^ and may cause environmental, ecological, and economic damage for many years.

**Figure 3:**
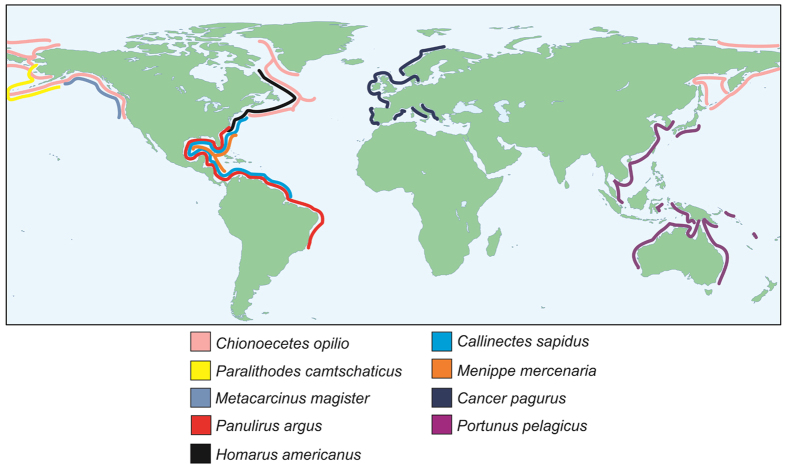
Global distribution of major crustacean pot and trap fisheries. Map created using Esri ArcGIS 10.0 ( http://www.esri.com/software/arcgis).

Total global landings from all crustacean trap fisheries grossing US $20 million or more annually (Fig. 3) average 615,560 MT and are worth US $2.5 billion (Table 1). Together, these high-value fisheries deploy tens of millions of pots and traps, millions of which become derelict each year. Extending findings from Chesapeake Bay blue crab to global crustacean fisheries suggests that removing less than 10% of the derelict pots and traps in these fisheries could increase landings by 293,929 MT, at a value of US $831 million annually. For blue crab in the United States, extensive removals from Atlantic and Gulf state fisheries might increase landings by over 40%, generating US $62 million in annual revenue benefits. In these and other pot and trap fisheries, substantial levels of gear loss likely lead to costly and inefficient outcomes. Net benefits of removal programs will ultimately depend upon removal costs however, which may vary widely.

## Discussion

Increases in severe weather, boating traffic, and gear conflicts, arising from continued climate change^27^ and global economic growth^28^,^29^, could intensify gear loss over the coming decades. Preventative measures which incentivize gear conservation have been advocated in place of widespread removals on the basis of cost-effectiveness and sustainability^11^,^26^. In deep-water fisheries utilizing heavy gear, derelict gear location and removal may remain cost prohibitive^30^. Here it was seen that removal efforts can be economically viable, generating harvest and revenue benefits in excess of program costs. Simple, low-cost, and easily enforceable preventative measures should be introduced when possible, however a “one size fits all” approach has been argued to be problematic^12^ and more research comparing cost-effectiveness of different measures is currently needed^26^. As all gear loss cannot be prevented, a combination of preventative and mitigating measures, such as the incorporation of effective biodegradable escape mechanisms^13^,^30^, together with removals that target areas of high fishing pressure, is likely to yield benefits superior to any individual strategy in isolation. For small-scale removal programs, removing derelict gear from areas which regularly experience intense effort is recommended.

The harvest enhancing effects of derelict gear removals explored here were entirely the result of reduced gear competition and improved efficiency. Other studies have found derelict gear to be a source of mortality for target and non-target species^10^,^11^,^12^,^13^,^14^,^15^,^16^, indicating the benefits of removals estimated here, though considerable, may be a lower bound. If removals led to a healthier and more abundant blue crab population, and this then led to harvest increases, total program benefits should increase. As crab and other crustaceans are generally attracted to bottom structure, and have been observed to regularly approach both active and derelict pots^19^,^20^,^21^,^22^,^23^, it is likely that the use of biodegradable escape mechanisms would reduce, though not eliminate, the efficiency reducing effects of derelict gear.

Improvements in crustacean harvests resulting from the removal or reduction of rival derelict pots and traps can be biologically sustainable and offer clear, unfettered economic benefits. In the removal program analyzed here, it is estimated that approximately 60 million additional crab were harvested over the program’s six years. This level of supplementary take averaged 2% of the estimated annual abundance, and throughout the removal program, commercial exploitation rates were found to be well within or below biological targets^31^. By 2012, blue crab abundance had increased 160% above 2008 estimates and a large number of juveniles were also observed. Following three seasons of intense removal efforts in which 80% of all removals occurred, there was no indication that the enhanced harvests afforded through derelict pot removals compromised blue crab recruitment or stock health. It is clear from our analysis however, that, absent the Virginia Marine Debris Location and Removal Program, the briefly bountiful blue crab would have yielded less harvest and economic benefit.

The economic costs of derelict gear examined here are likely not unique to pot and trap fisheries. Lost trammel-nets, gillnets, longlines, and bottom trawl gear pollute marine environments all over the world^11^,^26^ and attract target and non-target species in much the same way as derelict pots and traps^24^. In these fisheries, it might be expected that active gear is underproductive. In addition to lost harvests arising from stock depletion by ghost fishing derelict gear, and any other detrimental biological or ecological effects, competition with active gear may generate economic inefficiencies similar to those found for Chesapeake Bay blue crab.

The dilemma of derelict gear is, at its core, a common property problem. Assets which are owned by all are all too often of value to no one. The lost time, effort, and materials which result from needlessly inefficient gear represent a source of non-recoverable economic waste. These costs, though previously unacknowledged, are perhaps equally tragic to the ecological and environmental damage more commonly associated with derelict gear. Reducing or removing dominant sources of marine debris from the world’s oceans, bays, and estuaries is essential not only to restoring and protecting local ecologies and environments, but also to revitalizing resource dependent communities and cultures.

## Methods

### Chesapeake Bay

The Virginia Marine Debris Location and Removal Program employed commercial crabbers to locate and remove derelict fishing gear from Virginia tidal waters. Participants were assigned to broad areas according to anticipated derelict pot abundance, travel time, and other logistical considerations such that excessive overlap was avoided. Individuals were provided with a side imaging unit (Humminbird™ 1197SI side imaging unit, dual frequency 455–800 kHz) preprogrammed to scan using 23 m (75 ft) swaths and acquire GPS points (survey tracks) every 30 seconds. The date, time, and location (waypoint), as well as various item descriptors, were recorded for all retrieved pots. During the first four years of the program (2008–2012), 32,421 derelict blue crab, peeler, and eel pots were recovered. The last two years of the program saw an abbreviated removal program in which 1,987 derelict pots were removed.

There are approximately 300,000 pots licensed and fished in Virginia, 20% of which, or about 60,000, are lost each year^15^. Assuming half of all derelict pots completely degrade each year—a conservative assumption as structural integrity has been shown to last for at least two years^18^—Virginia’s “stock” of derelict pots can be described by the discrete time equation: 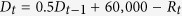, where *D*_*t*_ and *R*_*t*_ are the stock and removals of derelict pots in year *t*, respectively. Using this formulation, intense removal efforts during the first three years of the program would have decreased the standing stock of derelict pots by 15%. Targeted hotspot removals later in the program likely led to localized decreases, however, the total stock of derelict pots would have increased during this time. Over the program’s six years, removals are thought to have reduced the quantity of derelict pots by ~9% on average.

To investigate the impact of the removal program on the blue crab fishery, harvests were modeled using a modified Schaefer^32^ specification which included derelict pot removals:


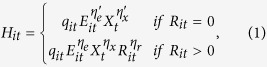


where *H*_*it*_ is the harvest in area *i* at time *t*; *q*_*it*_ is an area- and time-specific catchability coefficient; *E*_*it*_ is the effort in area *i* at time *t*; *X*_*t*_ is the stock at time *t*; *R*_*it*_ is the amount of derelict gear removed from area *i* at time *t*; and *η*_*e*_, *η*_*x*_, and *η*_*r*_ are elasticity parameters.

Data necessary to estimate equation (1) was acquired from several different sources. Annual blue crab harvests and effort (number of pots) from 1994–2014 for 54 management delineated fishing areas were obtained from the Virginia Marine Resources Commission, the state agency responsible for managing blue crab. The 34,408 derelict pot removals were then overlaid into georeferenced management areas using the Identity operation in ArcGIS 10.0 and matched by year to harvests and effort. High-quality stock abundance estimates, derived from an annual winter dredge survey which samples ~1,500 sites throughout the Chesapeake Bay^31^, were appended to harvest, effort, and derelict pot removal data. Equation (1) was estimated using a flexible transcendental logarithmic formulation which allowed for area random effects (see Supplementary Table S1).

Evaluating the impact of removals on harvests was accomplished through comparison of model predictions with and without derelict pot removals:


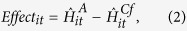


where 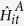 and 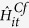
are harvest predictions from equation (1) given actual removals and a counterfactual of zero removals. *Effect*_*it*_ is the difference in predicted harvests for area *i* at time *t* attributable to the removal of derelict gear. Summation of equation (2) over *i* and *t* produced a measure of total program effects. Harvest effects were converted to revenues using average annual ex-vessel prices for Virginia hard shell blue crab in 2014 dollars.

While the potential for confounding bias in equation (2) cannot be totally eliminated, several aspects of the data and statistical model used reduce its likelihood. First, of the 54 management areas where harvest and effort were observed, 12 (22%) saw no removals during any year of the program. The number of areas experiencing removals in any given year averaged 32 (59%) and never exceeded 41 (76%). Overall, removals were found to exhibit a high degree of temporal and spatial variation (*cv*(*R*_*t*_) = 0.73, *cv*(*R*_*i*_) = 1.52), providing a rich set of data with which to identify marginal removal effects. Second, effort did not appear to respond to removals. That is, areas which saw more removals did not experience corresponding increases in effort. Were this not the case, a more complex counterfactual environment would be required to evaluate the removal program. Finally, the statistical harvest model included parameters to control for extraneous factors affecting harvests that were unrelated to the removal program. Area random effects enabled differences in catchability across areas to be modeled apart from any differences in area-specific removals, while a dummy indicator variable was included to control for exogenous shifts in catchability occurring contemporaneously with the removal program. Similar quasi-experimental empirical methods have been used to evaluate fisheries policies and isolate program effects in other contexts^33^,^34^ (see Supplementary Information for additional background and description of the data and harvest model).

### Global Analysis

To calculate the global impacts of wide-spread derelict gear removal or reduction, it was assumed that the following ratio would be maintained across crustacean pot and trap fisheries:


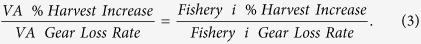


Rather, the increase in harvests which could be expected to result after removing derelict gear from the grounds of fishery *i*, in an amount proportionate to that removed through the Virginia Marine Debris Location and Removal Program (i.e., ~9%), would depend on the rate of gear loss in that fishery. This relationship might be expected as most crustacean fisheries utilize pots and traps constructed from similar materials and operate in near-shore coastal environments, suggesting similar rates of gear decay. Proportionate removals from a fishery with a high rate of gear loss would imply many pots and traps were removed, and thus a large harvest increase should be expected. Additionally, as removals from areas of high potting effort were found to be more effective at enhancing harvests, removal benefits should be greater in fisheries with large stocks of derelict gear experiencing significant production inefficiencies.

To predict harvest increases using the ratio (3), our estimate of a 27% increase in blue crab harvests in Virginia, where the gear loss rate has been found to be 20%, was applied to global landings and gear loss data (Table 1). Mean loss rates were used for those fisheries where a range was reported, while a conservative 20% was applied to three fisheries without gear loss rate measurements (snow crab *Chionoecetes opilio*, edible crab *Cancer pagurus*, and stone crab *Menippe mercenaria*). Average prices were used to calculate revenues^35^,^36^,^37^. Large increases in landings could have offsetting price effects, however, due to data limitations, this possibility was not investigated here. Additionally, as multiple commercial fisheries exist for each of the included species, overall gear loss rates may differ from those used here. Differences in habitat and gear across fisheries may affect results, though attraction to bottom structure is a commonly observed crustacean behavior^19^,^20^,^21^,^22^,^23^ and removal of derelict gear from global crustacean fisheries could hold similar efficiency improving effects to those observed for Chesapeake Bay blue crab if animals attracted to derelict gear might otherwise be caught by actively fished gear.

## Additional Information

**How to cite this article**: Scheld, A. M. *et al.* The Dilemma of Derelict Gear. *Sci. Rep.* **6**, 19671; doi: 10.1038/srep19671 (2016).

## Supplementary Material

Supplementary Information

## Figures and Tables

**Figure 1:**
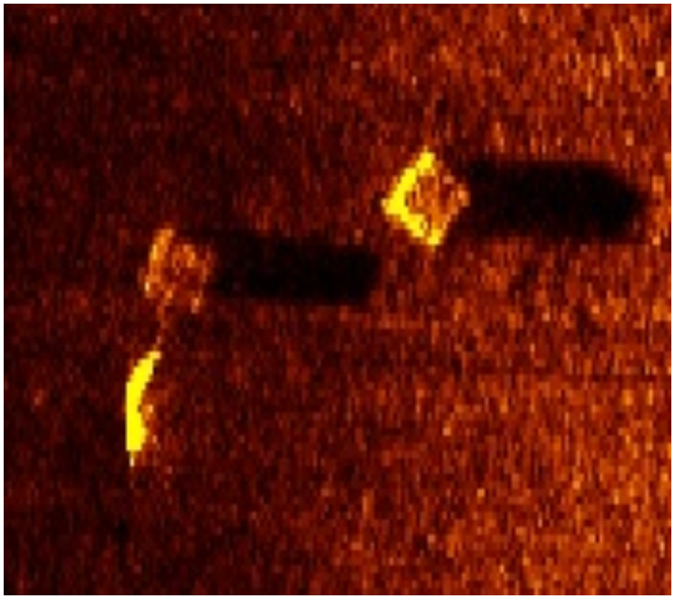
Side-scan sonar image of active/buoyed (left) and derelict (right) crab pots in the Chesapeake Bay (credit: CCRM/VIMS).

**Table 1:** Gear loss and global landings for major crustacean pot and trap fisheries.

Species	Annual Gear Loss (% Deployed)*	Landings (MT)	Revenues (US$)	Major Producers
Blue swimmer crab *Portunus pelagicus*	70	173,647	$199M†	China, Philippines, Indonesia, Thailand, Vietnam
American lobster *Homarus americanus*	20–25	100,837	$948M	Canada, USA
Blue crab *Callinectes sapidus*	10–50	98,418	$152M	USA
Queen crab/snow crab *Chionoecetes opilio*	NA	113,709	$401M	Canada, St. Pierre and Miquelon (France), USA
Edible crab *Cancer pagurus*	NA	45,783	$49M‡	United Kingdom, Ireland, Norway, France
Dungeness crab *Metacarcinus magister*	11	35,659	$169M	USA, Canada
Spiny lobster *Panulirus argus*	10–28	34,868	$500M§	Bahamas, Brazil, Cuba, Nicaragua, Honduras, USA
King crab *Paralithodes camtschaticus*	10	10,137	$99M	USA
Stone crab *Menippe mercenaria*||	NA	2,502	$24M	USA
TOTAL		615,560	$2.5B	

Average MT and US $ 2003–2012. Data from: NOAA Office of Science and Technology, National Marine Fisheries Service, Commercial fisheries statistics http://www.st.nmfs.noaa.gov/st1/commercial/index.html; Food and Agriculture Organization, United Nations, Fisheries and Aquaculture Department, http://www.fao.org/fishery/search/en, Fisheries and Oceans Canada http://www.dfo-mpo.gc.ca/stats/commercial/sea-maritimes-eng.htm.

^*^Estimates from Bilkovic *et al.* (2012).

^†^Based on an average price of US $1.15/kg (35).

^‡^Based on 2004–2012 average price of US $1.07/kg (36).

^§^See (37).

^||^Claws only.
