# Supplementary material for: The Dilemma of Derelict Gear
Source: Sci Rep. 2016 Jan 21;6:19671. doi: 10.1038/srep19671 (PMC4726330; doi:10.1038/srep19671)
Supplement: Supplementary Information [file srep19671-s1.pdf]

Supplementary Information for: The Dilemma of Derelict Gear

A.M. Scheld, D.M. Bilkovic, K.J. Havens

Background

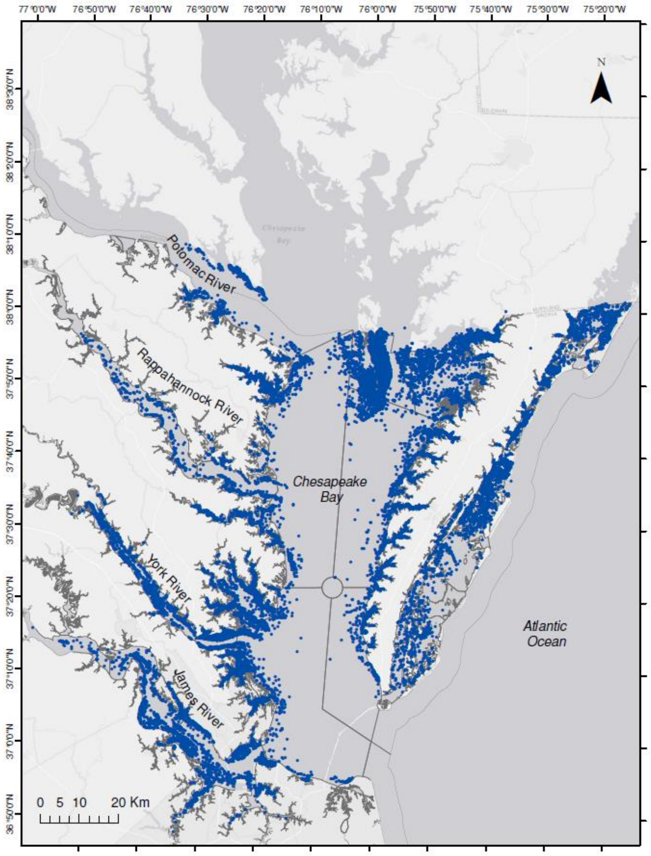

Figure S1 | Map of derelict pot removals. Dark grey lines define management area boundaries. The main stem of the bay is closed to all harvests. Map created using Esri ArcGIS 10.0 (http://www.esri.com/software/arcgis).

From 1994 until 2008, Virginia blue crab harvests and effort largely declined as managers tightened effort restrictions in response to poor stock conditions. During this period, harvest per pot averaged 0.75 kg/pot (*SD* = 0.24). Beginning in the 2008, the Virginia Marine Debris

Location and Removal Program funded commercial crabbers during the winter closed fishing season to find and remove derelict fishing gear. Over six consecutive winters, from 2008-2014, 34,408 derelict pots were removed (Fig. S1).

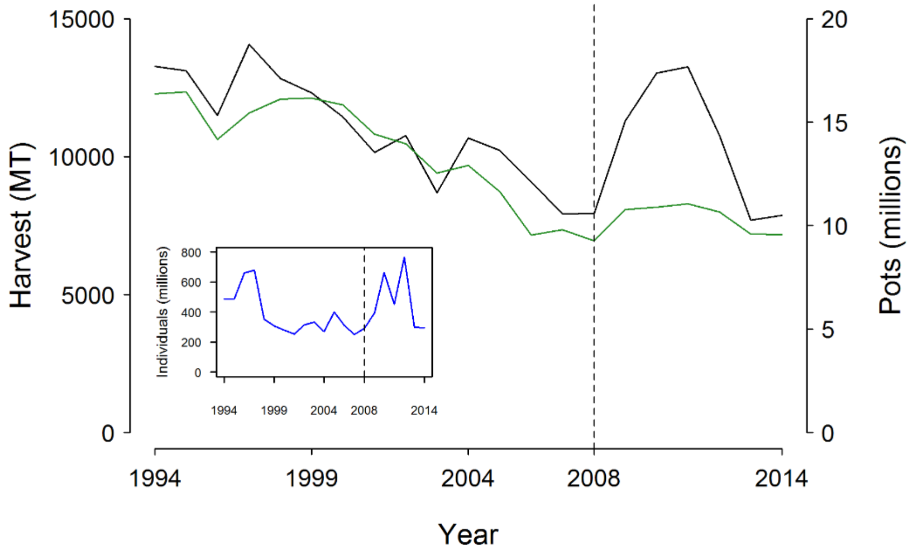

Figure S2 | Annual Virginia blue crab pot fishery. Harvests shown in black and effort in green. Inset figure plots annual Chesapeake Bay estimated blue crab abundance. Dashed lines at first year of the Virginia Marine Debris Location and Removal Program. The open crabbing season lasted from April 1st through the end of November in all years except 2014, when the season was extended by two weeks.

From the onset of the removal program, harvests and gear efficiency were seen to increase dramatically (Fig. S2). During the program's first three years, when over 80% of all removals occurred, harvest per pot increased to 0.97 kg/pot (SD = 0.31), indicating each fished pot was yielding an additional blue crab on average (1 crab ≈ 0.22 kg). As removal efforts declined, so too did harvests and gear efficiency, returning to pre-removal levels during the last three years of the program (harvest per pot: M = 0.76 kg, SD = 0.26).

Annual blue crab abundance estimates showed considerable stock improvements contemporaneous with derelict pot removal efforts. While it was anticipated that removal of derelict gear would be biologically beneficial, concurrent closure of the controversial winter dredge fishery, which primarily targets females as they lay dormant in the lower bay, complicates identification of the program's biological effects. Additionally, though it might be expected that the removal of derelict pots would enhance harvests and gear efficiency due to the reduction of rival ghost fishing gear, this conclusion is confounded by abundance increases which would also be expected to enhance harvests and gear efficiency. To separate out the effects of changes in abundance, effort, and derelict gear removals on harvests, it is necessary to first construct a model of blue crab production.

Model Specification

A flexible Schaefer harvest function can be written as:

(S1) $$H_{it} = q_{it} E_{it}^{\eta_e} X_t^{\eta_x},$$

where H_{it} is the harvest in area i at time t; q_{it} is an area- and time-specific catchability coefficient; E_{it} is the effort in area i at time t; X_t is the stock at time t; and η_e and η_x are effort and stock elasticity parameters. The harvest function (S1) allows harvests to vary spatially with effort, temporally with effort and stock, and also allows for area- and time-specific shifts in catchability. The inclusion of elasticity parameters enables a flexible harvest response to both effort and stock. To model the effects of derelict gear removals, equation (S1) was modified to include the amount of derelict gear removed from area i at time t (see Methods).

Data

The Virginia Marine Resources Commission (VMRC) requires fishers to submit weekly reports which specify total pots (and other gear) fished, their location, and pounds of blue crab harvested. From these weekly reports, aggregate annual data on area-specific harvest and potting effort from 1994-2014 for 43 unique management areas and 11 area-aggregates was obtained. Harvest and effort in reporting areas which were visited by fewer than three individuals in a given year were aggregated by the VMRC to a higher spatial level (river system) to maintain confidentiality. Approximately 10% of harvests occurred in 11 spatial aggregations representing 31 separate management reporting areas. There were two management areas with no recorded harvests or effort.

The final dataset consisted of 1,058 observations of annual harvest and effort from 43 management areas (n = 903) and 11 area aggregates (n = 155); 21 annual observations of estimated blue crab abundance; and 286 annual area removal observations. Abundance and removal observations were matched to harvests and effort by year and area-year, respectively. Though all included areas (and area-aggregates) experienced effort and harvest during the removal program, over 20% saw no derelict pots removed. Areas and times which experienced no removals were coded as “0”. The resulting panel of data was slightly unbalanced as some management areas, included in the area aggregates, were infrequently visited. Balancing the panel did not change model parameter estimates, results, or general conclusions.

Annual price data was obtained from the National Oceanic and Atmospheric Administration’s Office of Science and Technology, who maintain an updated online database of commercial fisheries landings, searchable by state.

Econometric Estimation

To estimate equation (1), a transcendental logarithmic formulation was employed:

(S2)
$$\ln H_{it} = \beta_0 + \beta_1 \ln E_{it} + \beta_2 \ln X_t + \beta_3 \ln R_{it} + \beta_4 \ln E_{it} \ln E_{it} + \beta_5 \ln X_t \ln X_t + \beta_6 \ln R_{it} \ln R_{it} + \beta_7 \ln E_{it} \ln X_t + \beta_8 \ln E_{it} \ln R_{it} + \beta_9 \ln X_t \ln R_{it} + \beta_{10} I_t + \alpha_i + \varepsilon_{it},$$

where H_{it} is the kg of blue crab harvest in management area i and year t; E_{it} is the number of blue crab pots fished in management area i and year t; X_t is an estimate of blue crab abundance in year t; R_{it} is the number of derelict pots removed from area i in year t (occurring before effort and harvest of year t but subsequent to that of year t-1); I_t is an indicator function which equals one during years of the removal program and zero otherwise; α_i is an area specific effect; and ε_{it} is a normally distributed random error term. Before estimation, one unit was added to all derelict pot removal observations.

The catchability and elasticity parameters of equation (1) can be derived from parameters in the specification (S2) as:

(S3a)
$$q_{it} = \exp\left(\beta_0 + \beta_{10} I_t + \alpha_i\right);$$

(S3b)
$$\eta_e = \beta_1 + \beta_4 \ln E_{it} + \beta_7 \ln X_t + \beta_8 \ln R_{it};$$

(S3c)
$$\eta_x = \beta_2 + \beta_5 \ln X_t + \beta_7 \ln E_{it} + \beta_9 \ln R_{it};$$

(S3d)
$$\eta_r = \beta_3 + \beta_6 \ln R_{it} + \beta_8 \ln E_{it} + \beta_9 \ln X_t.$$

In equation (S3a), the catchability coefficient of equation (1) is shown to vary by area due to the inclusion of α_i, an area specific effect. The indicator variable I_t allows for a shift in catchability

occurring contemporaneously with, but unrelated to, derelict pot removals. Equations (S3b-d) show that elasticity parameters from the harvest function (1) are modeled to be extremely flexible, permitted to change in response to values of included independent variables.

Statistical analyses and estimation were done in R[38]. Equation (S2) was initially estimated in both fixed and random effects frameworks (i.e., in separate specifications α_i, the individual area effect, was modeled as resulting from fixed, non-random processes as well as random factors). A Hausman test indicated the individual area effects (α_i's) were not correlated with the model's independent variables ($H = 14.99 \sim \chi^2(10)$, $p = 0.13$), implying a random effects specification was both consistent and efficient. The final random effects model was fit using lmer in the lme4 package[39]. A large amount of the variance in harvests was explained through the model's fixed factors (marginal $R^2 = 0.942$), while area random effects explained a lesser amount (conditional $R^2 = 0.974$)[40]. The strong fit suggests equation (S2) does well in explaining the harvest process, which is largely determined through effort, stock, and, when applicable, derelict gear removals. A residual bootstrap procedure, also contained in the lme4 package, was used to sample 10,000 parameter vectors. During bootstrap sampling, random effects were held fixed at the original model estimates, i.e., only residuals were resampled in constructing synthetic observations.

Table S1 | Mean bootstrapped parameter estimates. Elasticities calculated at the mean of all variables. n = 10,000 bootstrap samples. Parameter significance: *** 0.01; ** 0.05; * 0.1.

Parameter (Model)	Estimate (SE)
β_0 (S2)	-10.794 (3.120)***
β_1 (S2)	1.202 (0.144)***
β_2 (S2)	3.145 (0.953)***
β_3 (S2)	-0.133 (0.101)
β_4 (S2)	0.001 (0.005)
β_5 (S2)	-0.210 (0.077)***
β_6 (S2)	-0.003 (0.004)
β_7 (S2)	-0.035 (0.016)**
β_8 (S2)	0.008 (0.004)*
β_9 (S2)	0.017 (0.014)
β_{10} (S2)	-0.044 (0.023)**
η_e (1)	1.026 (0.048)***
η_x (1)	1.500 (0.489)***
η_r (1)	0.054 (0.010)***

All harvest function (equation 1) production elasticities were found to be positive and statistically significant (Table S1). The mean values of η_e and η_x suggest increases in both factors led to more than proportionate percentage increases in harvest, though neither elasticity was greater than one at a 95% confidence level. A strictly positive bootstrapped distribution of η_r indicated that harvests increased significantly in response to derelict pot removals, supporting the hypothesis that derelict gear reduces harvests. Catchability (equation S3a) varied by area due to the inclusion of area random effects, though this variation was relatively minor ($cv(q_{it})$ = 0.23). Additionally, catchability was seen to decrease by 4% during the removal program as a result of factors unrelated to removals, effort, or stock abundance. This suggests that the Virginia Marine Debris Location and Removal Program occurred contemporaneously with poor environmental conditions or other factors not conducive to blue crab harvest.

Program Evaluation

To uncover the effects of the Virginia Marine Debris Location and Removal Program on harvests and gear efficiency, the empirical harvest model (S2) was used to generate predictions under two different scenarios: actual removals and zero removals. In the zero removals counterfactual, all removal observations were set to zero before predicting harvests (note that one unit was added to all removals before model estimation; in the counterfactual, all removal observations equaled one, the natural logarithm of which is zero).

During counterfactual estimation, the values of effort and stock remained fixed. Thus, the counterfactual comparison was between harvests with and without derelict gear removals, assuming effort and stock were not directly affected by the program. This simple hypothetical is justifiable for several reasons. First, there is a high degree of site fidelity, habitual behavior, and territoriality among Virginia crabbers. A regression of the number of active pots per area and year on removals, controlling for constant effort differences between areas and increased total effort during the program's first three years, yielded an insignificant result ($p = 0.31$). This finding indicates that effort did not respond directly to the level of removals, and therefore it is valid to use observed effort in the counterfactual. Second, though removal of derelict gear benefited the blue crab stock by reducing ghost fishing mortality, contemporaneous changes in bay-wide population abundance were likely the result of management measures and environmental conditions. Total estimated abundance increased by 160% from 2008 to 2012 and then dropped sharply by more than 60% over the next two years. During the removal program, estimated abundance varied by 44% year-to-year on average. This level of population change is far beyond that which might be expected to result from removing 9% of derelict gear. Finally,

aside from the removal of rival gear, which might reduce efficiency of active gear, there was no indication that the program significantly affected other variables potentially related to harvests.

Sensitivity Analysis

Concerns surrounding effort reporting accuracy were addressed through sensitivity analyses. The econometric specification of harvest (equation S2) utilized log transformations for all variables, thus relationships were evaluated in terms of percentage changes and not actual values. Constant effort misreporting would therefore have no effect on model results (e.g., if harvesters always used 50% more pots than reported, predicted program effects would remain unchanged). Variable or irregular misreporting, where data on potting effort in certain areas or at certain times is inaccurate, could affect parameter estimates, model results, and general conclusions however.

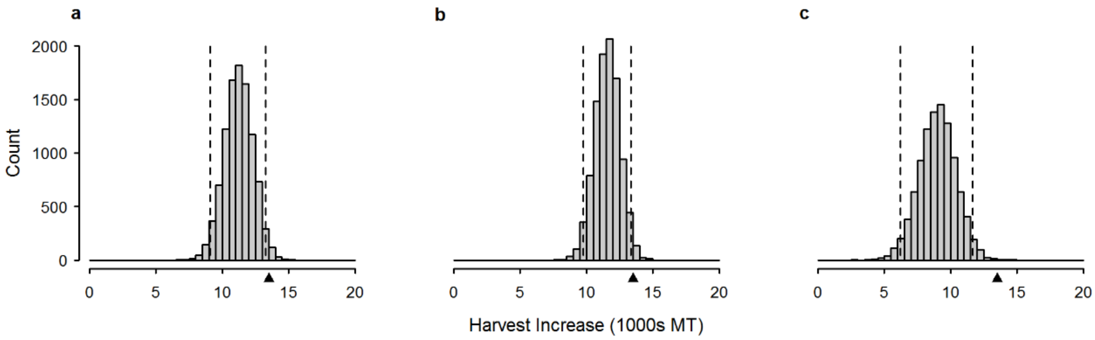

Figure S3 | Sensitivity analysis of program effects. Three alternative scenarios were considered (*n* = 10,000 simulations under each scenario): a) half of all observations underreport actual effort by 50% ; b) three-quarters of all observations underreport actual effort by 50% ; and c) one-quarter of all observations underreport effort by 50% while one-quarter of all observations overreport effort by 50%. Dashed lines specify 95% confidence interval and black triangle is placed at mean effects from the null model (zero misreporting). Bin width is 500 MT.

To evaluate the impact of variable effort misreporting on the predicted effects of derelict gear removal, three alternative scenarios were investigated: 1) half of all observations

underreport actual effort by 50%; 2) three-quarters of all observations underreport actual effort by 50%; and 3) one-quarter of all observations underreport effort by 50% while one-quarter of all observations overreport effort by 50%. The range of scenarios considered allowed for both under- and overreporting, though managers indicated that underreporting of effort is likely more commonplace (Rob O'Reilly, personal communication). For each scenario, those observations considered to be misreporting effort were randomly selected from the pool all observations according to scenario-specific probabilities of under- and overreporting. The number of pots reported in selected misreporting observations were then rescaled to reflect under- or overreporting and all models were re-estimated. This process was repeated 10,000 times for each of the three scenarios, storing mean predicted program effects following model estimation at each iteration (Fig. S3).

In all cases, variable misreporting tended to decrease mean effects. If misreporting is occurring, the degree of upward bias in program effects estimated by the null model (zero misreporting) would depend on the variance in measurement error. Highly variable misreporting (both in magnitude and direction) implies observed/recorded effort is a poor proxy for actual effort and the strong fit of equation (S2) is reduced considerably. The effects of derelict gear removal are then reduced as removals were found to be significantly more effective in areas and years of high potting effort. Fortunately, there is little reason to believe misreporting is highly variable. It is more likely that effort is consistently underreported, the effects of which appear to be minimal. In all scenarios considered, harvest improvements remain large and positive despite variable effort misreporting. General results and conclusions surrounding the effects of derelict gear removals on harvests are therefore considerably robust to misreporting of effort.

References

38. R Core Team. R: A Language and Environment for Statistical Computing. (R Foundation for Statistical Computing; Vienna, Austria, 2013; http://www.R-project.org/).
39. Bates, D., Maechler, M., Bolker, B. & Walker, S. lme4: Linear mixed-effects models using Eigen and S4. (R Package Version 10-5, 2014; http://CRAN.R-project.org/package=lme4).
40. Nakagawa, S. & Schielzeth, H. A general and simple method for obtaining R2 from generalized linear mixed-effects models. *Methods in Ecology and Evolution* **4**, 133-142 (2013).
